# Insights into Actin-Myosin Interactions within Muscle from 3D Electron Microscopy

**DOI:** 10.3390/ijms20071703

**Published:** 2019-04-05

**Authors:** Kenneth A. Taylor, Hamidreza Rahmani, Robert J. Edwards, Michael K. Reedy

**Affiliations:** 1 Institute of Molecular Biophysics, Florida State University, Tallahassee, FL 32306-4380, USA; hr13b@my.fsu.edu; 2 Department of Cell Biology, Duke University Medical Center, Durham, NC 27607, USA; rj.perz-edwards@duke.edu (R.J.E.); michael.reedy@duke.edu (M.K.R.)

**Keywords:** striated muscle, image reconstruction, muscle physiology

## Abstract

Much has been learned about the interaction between myosin and actin through biochemistry, in vitro motility assays and cryo-electron microscopy (cryoEM) of F-actin, decorated with myosin heads. Comparatively less is known about actin-myosin interactions within the filament lattice of muscle, where myosin heads function as independent force generators and thus most measurements report an average signal from multiple biochemical and mechanical states. All of the 3D imaging by electron microscopy (EM) that has revealed the interplay of the regular array of actin subunits and myosin heads within the filament lattice has been accomplished using the flight muscle of the large water bug *Lethocerus* sp. The *Lethocerus* flight muscle possesses a particularly favorable filament arrangement that enables all the myosin cross-bridges contacting the actin filament to be visualized in a thin section. This review covers the history of this effort and the progress toward visualizing the complex set of conformational changes that myosin heads make when binding to actin in several static states, as well as the fast frozen actively contracting muscle. The efforts have revealed a consistent pattern of changes to the myosin head structures as determined by X-ray crystallography needed to explain the structure of the different actomyosin interactions observed in situ.

## 1. Introduction

**Figure 1:**
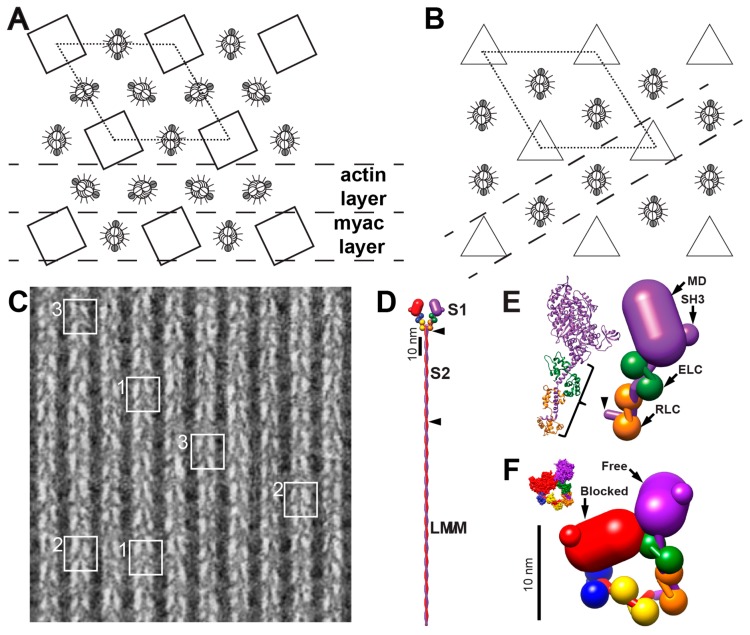
Introduction. (**A**) The filament lattice of the *Lethocerus* flight muscle. Thick filaments (squares) have four myosin molecules per 14.5 nm axial repeat (crown); crowns of individual filaments at any level are rotationally aligned. Thin filaments are placed midway between thick filaments with the troponin (Tn) complex oriented approximately perpendicular to the inter-filament axis of the hexagonal unit cell (dotted line). The filament placement permits two types of 25 nm section to be cut from the lattice: The actin layer containing only thin filaments and the myac layer containing alternating thick and thin filaments; (**B**) a simple vertebrate striated muscle lattice typically found in teleost fishes, has thick filaments, with three myosin molecules per 14.3 nm level placed at the corners of the unit cell (dotted line) but with thin filaments placed at trigonal positions [15]. The thin filaments are shown in rotational register consistent with observation [16]. No thin section comparable to those obtained from the *Lethocerus* flight muscle can be cut from the vertebrate striated muscle. The section closest in composition to a myac layer is marked with the dashed line. Note that, unlike the *Lethocerus* myac layer, myosin heads can approach the thin filaments from out-of-plane positions; (**C**) a myac layer thin section from rigor flight muscle. The myosin cross-bridges are distributed among classic double chevron motifs (white boxes labeled 1) consisting of paired lead bridges and paired rear bridges, single chevron motifs (white boxes labeled 2) consisting of only paired lead bridges and incomplete double chevrons (white boxes labeled 3) with a single rear bridge. Most descriptions of myosin head distribution in other states of the *Lethocerus* flight muscle are described as departures from the rigor double chevron motif; (**D**) schematic diagram of a myosin dimer with heavy chains colored red and purple. Paired myosin heads are positioned at the N-terminus of a ~160 nm coiled coil, the myosin rod, which can be proteolytically cleaved into a segment containing about 2/3 of the rod dubbed light meromyosin or LMM and a segment dubbed S2 containing the other 1/3; (**E**) the myosin head shown both as a ribbon diagram and schematically, containing a motor domain (MD, purple), essential light chain (ELC, green) and regulatory light chain (RLC, orange); (**F**) the Interacting Heads Motif (IHM) with the blocked head (red) positioned on the free head (purple). D–F from reference [6].

Electron microscopy (EM) of the flight muscles of large water bugs of the *Lethocerus* genus has informed the evolution of models of muscle contraction since Reedy, Holmes and Tregear showed pronounced changes in the axial orientation of myosin heads in rigor flight muscle when ATP is added [1]. This observation formed part of the basis of the swinging cross-bridge model of muscle contraction [2]. What makes the *Lethocerus* flight muscle attractive for structural work is its high degree of order when compared to the vertebrate striated muscle and its favorable filament arrangement, which contributes in no small way to the former. Both striated muscles have a hexagonal array of thick filaments surrounded by six thin filaments (Figure 1A,B). However, in *Lethocerus* the thin filaments are placed at diad positions in the unit cell, which are located midway between pairs of thick filaments. In the vertebrate striated muscle, thin filaments are located at triad positions, which are centered between triplets of thick filaments (Figure 1B). The thin filament has approximate 2-fold symmetry, so placement at a diad position facilitates a more symmetrical attachment of cross-bridges than would occur from three symmetrically placed thick filaments. The filament arrangement in *Lethocerus* also makes it possible to cut thin sections of plastic embedded muscle that contain single layers of alternating thick and thin filaments, the so-called “myac” layer (Figure 1C). Only the two neighboring thick filaments contribute myosin-head attachments to the intervening thin filament within the myac layer. The great advantage of the myac layer thin sections in the *Lethocerus* flight muscle was the clear view provided of all the myosin heads attaching the thin filament from which patterns and quality of preservation could be evaluated.

In addition to its well-ordered filament placement, the *Lethocerus* flight muscle also has modified filament periodicities that contribute to its high degree of order within sarcomeres. Unlike the thin filaments of the vertebrate striated muscle, which have an actin helical symmetry of close to 13/6 subunits/turn [3], the thin filaments of the *Lethocerus* flight muscle have a symmetry of 28/13 subunits/turn [4]. Consequently, the actin symmetry and the troponin (Tn)/tropomyosin (TM) symmetry are congruent. A rise of seven actin subunits along the long pitch helix results in the Tn complex rotating 180°; all the Tn/TM regulatory units are identically oriented azimuthally with respect to the inter-thick-filament axis while following a left-handed helix with a rise of 12.9 nm and a rotation of −60° about the thick filament axis [5]. In striated muscles of vertebrates with 13/6 symmetry, the Tn/TM units define a super helix that rotates ~14° every seven actin subunits along the actin’s long pitch helix resulting in a systemic variation of Tn sites with respect to the inter-filament axis. The combination of mismatch between the thick and thin filament axial periodicities, Tn/TM super helical symmetry and thin filament placement at trigonal positions within the lattice makes for a more chaotic arrangement of actin-myosin interactions. 

Myosin molecules comprise a pair of heavy chains which have a folded N-terminus containing the actin binding and ATPase activities followed by a long α-helical domain the first part of which binds two different light chains, the essential light chain or ELC and the regulatory light chain or RLC (Figure 1D,E). Each light chain binds to the initial segment of α-helix to form the myosin lever arm, axial movements of which produce sarcomere shortening during muscle contraction. The remainder of the heavy chain forms an α-helical coiled-coil rod domain (Figure 1D). In the *Lethocerus* myosin filaments, ~150 nm of the rod is tightly bound within the filament backbone [6].

The axial spacing between levels of myosin heads on the thick filament of invertebrate striated muscles including *Lethocerus* is 14.5 nm [7,8]. These levels are referred to as “crowns” [9] because the myosin head density in the relaxed *Lethocerus* flight muscle extends mostly perpendicular to the thick filament. Crowns of *Lethocerus* thick filaments have 4-fold, rotational symmetry in the A-band with successive crowns rotated +33.75° (right-handed) [10]. The lateral order between myosin head origins of thick filaments is precise even in the relaxed muscle; at any axial level within the lattice, the crowns on adjacent filaments are aligned both laterally and helically [11] even when thick filaments are offset axially by steps of one crown. Myac layers of rigor muscle show a well-defined repeating structure dubbed the “double chevron” (Figure 1C) [12]. The ability to cut 25–30 nm myac layer thin sections from *Lethocerus* flight muscle fibers made it the dominant model system for imaging actin-myosin interactions in situ.

The flight muscles of most modern insects have refined to a high degree a phenomenon known as stretch activation, which enables them to beat their wings rapidly at a submaximal calcium concentration, thereby obviating the requirement to lower calcium concentrations to <10^−6^ in order to relax between shortening cycles [13]. The effect of calcium on *Lethocerus* as well as the *Drosophila* flight muscle is complicated due to the presence of two TnC isoforms, the Tn component that actually binds Ca^2+^. The F1 isoform of TnC is needed to support stretch activated contractions, while the other isoform, F2, is needed for isometric contractions [14].

A key feature of stretch activated contractions is the phenomenon of shortening deactivation. Because [Ca^2+^] remains relatively constant during flight, shortening must terminate via some mechanism other than thin filament deactivation. Stretch activation is defined as a delayed development of active tension following a stretch. Stretch activation is most refined in well-ordered striated muscles that are stiff to sarcomere extension, such as asynchronous insect flight muscle and cardiac muscles of vertebrates, but can be observed to a lesser extent in the much less stiff vertebrate skeletal muscles [13]. The structural features that contribute to stretch activation are not completely described, but are likely to consist of multiple entities.

**Figure 2:**
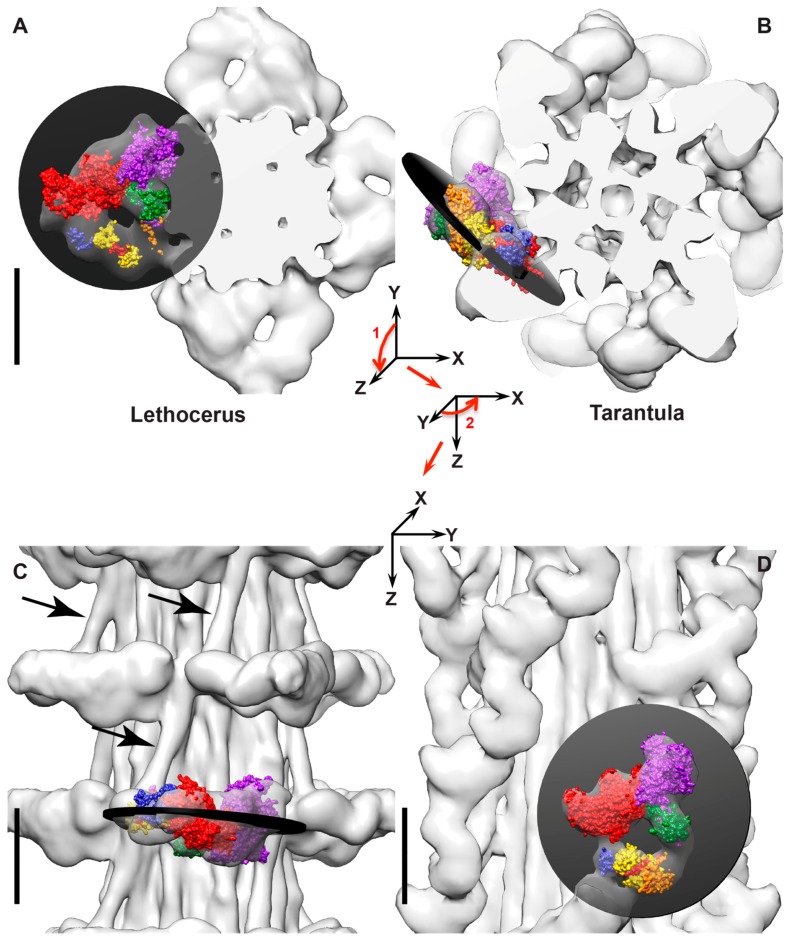
Orientation of the IHM in relaxed thick filaments. Coloring scheme the same as Figure 1F. (**A**,**C**) *Lethocerus* thick filament with PDB 1I84 fit into the map [6]. Note how the free head is placed tangentially against the filament backbone and the blocked head extends out into solvent space. (**B**,**D**) Tarantula thick filament with PDB 3JBH [17]. The IHMs can be approximated as flat planes, indicated by the black disks. Arrows in (**C**) indicate the position of the proximal S2 which is bent towards the left. The IHM of the *Lethocerus* flight muscle is approximately perpendicular to the filament axis; whereas the tarantula IHM is approximately tangential to the backbone surface. The angle between the plane of the *Lethocerus* IHM and the plane of the tarantula IHM is 91.6°. The axes in (**A**,**B**) indicate the initial M-ward cross-section view and the two 90° rotations, indicated by the red arrows, needed to transform to the longitudinal view shown in (**C**,**D**). Scale bars = 10 nm. Adapted from [6].

The thick filament structure of most muscles is relatively poorly understood in comparison to their thin filament structure. Cryo-electron microscopy (cryoEM) is changing that. Image reconstructions of relaxed thick filaments from the striated muscle of several species [17,18,19] including the *Lethocerus* flight muscle [6] have all shown the myosin heads folded into an asymmetric arrangement, first observed for smooth muscle myosin [20] and now called the Interacting Heads Motif (IHM, Figure 1F). One head within the IHM, the “blocked” head, is sterically inhibited from binding actin by its interaction with the other head, the “free” head, whose actin binding interface is not likewise inhibited and could potentially bind actin. For myosin to form the IHM, ATP must be cleaved to ADP•Pi with the Pi retained on the myosin head and the lever arm reoriented to the pre-powerstroke position characteristic of the transition state [20]. The *Lethocerus* thick filament structure is unique among those from striated muscles determined so far [6]. The *Lethocerus* IHM is oriented perpendicular to the thick filament axis with the free head interacting with the thick filament backbone (Figure 2A,C). In *Lethocerus*, the blocked head interacts only with the free head; the blocked head has no other intermolecular contacts. Free head contacts with the thick filament backbone are mostly through its RLC. In *Lethocerus*, the S2 is angled 17° azimuthally relative to the thick filament axis thereby fixing the pivot point of the blocked head to the S1–S2 junction. All the other relaxed thick filament structures have the IHM roughly tangential to the filament backbone with the blocked head contacting the proximal S2 (Figure 2B,D). With a relaxed thick filament structure in hand, as well as crystal structures of the myosin head in different steps of the catalytic cycle, some of the earlier interpretations of actomyosin interactions in the *Lethocerus* flight muscle made before this information was available can now be re-examined. 

The high resolution structure of the relaxed *Lethocerus* thick filaments [6] indicates that on filaments, the heavy chain that forms the free and blocked heads is predetermined. The thick filament backbone forces asymmetry into the environment of the two heads. For the free head and blocked head to exchange roles requires introducing a 180° change in twist into the exposed 11 nm segment of the S2 either adding or removing a full half turn. The rest of the S2 is held within the thick filament backbone. The high degree of order in the proximal S2 observed in the reconstructions [6] argues strongly against this possibility because averaging two structures that differ by a complete half turn of the coiled coil is incompatible with the high resolution obtained. This may also be true for thick filaments from other species.

This review concentrates on the visualization of actin-myosin interactions within the *Lethocerus* flight muscle because that work dominates the literature on 3D imaging of actin-myosin interactions in situ. Where work on a different muscle type is mentioned, the specific species and muscle will be defined. The early EM work on the *Lethocerus* flight muscle involved interpretations of projection images from thin sections of single filament layers as well as transverse sections. With the development of 3D image reconstruction techniques in the early 1970s, particularly techniques that explicitly utilized 2D spatial periodicity, i.e., 2D crystals, reconstructions of muscle sections began to show the overall shape of myosin attachments to actin in 3D (Table 1). However, because of certain types of intrinsic disorder present in the otherwise well-ordered sarcomeres these images did not resolve the individual myosin heads or actin subunits. Electron tomography (ET) produced more informative 3D images without spatial averaging, but with a significant noise component, making direct interpretation difficult. The later development of subvolume classification and averaging provided the most detailed images of myosin heads operating in situ in the muscle. Certain features of the actin-myosin interaction, particularly those indicating large azimuthal changes in the lever arm orientation relative to crystal structures of myosin heads, have been remarkably consistent among the many structures reported over the years.

## 2. Electron Microscopy of Thin Sections

### 2.1. Preparation of Muscle Tissue for 3D Electron Microscopy

The supply of *Lethocerus* is seasonal. Unless muscle tissue can be dissected and used immediately, it is generally glycerinated [35]. Muscle fibers and even individual myofibrils are too thick for examination in an EM, so most work on muscle tissue involves thin section EM. Production of thin sections involves the steps of fixation, embedding, sectioning followed by staining of the individual sections. Actin filaments are difficult to fix within muscle tissue because of their susceptibility to disruption by glutaraldehyde [36] and osmium [37], the conventional fixatives used for this purpose. Osmium tetroxide is also a popular fixative for freeze substitution. To avoid this problem, Michael and Mary Reedy developed a fixative combining tannic acid and uranyl acetate, dubbed TAURAC, which avoided both glutaraldehyde and osmium treatments and proved to be compatible with freeze substitution [35]. Specimens prepared using TAURAC for the first time showed the helical structure of actin in X-ray fiber diffraction patterns of embedded muscle. The development of a freeze substitution procedure using TAURAC opened the way to rapidly freezing active muscle for 3D visualization of myosin heads in action within the muscle lattice [35].

### 2.2. Methods for 3D Imaging of Muscle Tissue

In the early 1980s, the high degree of order within myac layer thin sections of the *Lethocerus* flight muscle suggested that 3D images could be obtained if the muscle lattice was treated as if it were a 2D protein array and images processed using methods developed for 2D crystals such as bacteriorhodopsin [38]. We refer to this approach as spatial averaging since the criterion used to decide if the repeating motifs (unit cells) can be averaged depends solely on their being periodically arranged within a lattice. Reconstructions obtained from tilt series always had some data missing due to physical limitation that prevented tilting to angles exceeding ~70°. The missing data is commonly referred to as a missing wedge, cone or pyramid depending on the shape of the missing data. A single axis tilt series results in a missing wedge; a dual axis tilt series results in a missing pyramid of data. At the time, spatial averaging was the singular option for 3D imaging for this type of specimen. 

Crowther and Luther [23] developed a second spatial averaging option, the oblique section image reconstruction (OSR), applicable to a specimen with a large unit cell for which sections could be cut substantially thinner than the unit cell dimensions. Sections cut oblique to the muscle lattice are usually the default; cutting well-oriented myac layers (e.g., Figure 1C) needed for 2D spatial averaging was much more difficult. Oblique sections 12–25 nm thick could be readily cut through the *Lethocerus* flight muscle fibrils, which have a hexagonal filament lattice of dimension 52 × 52 × 116 nm. The OSR was perfectly suited to 3D imaging of the *Lethocerus* flight muscle because it provided an average image of a complete unit cell, not just a single filament array, with no missing wedge. In some cases, the Fourier transform of the OSR could be compared with the X-ray diagram of the unfixed (native) muscle. OSR was developed to its greatest extent using imaging of the *Lethocerus* flight muscle as the driving biological problem [11,26,28,39,40]. However, electron tomography (ET) with its ability to 3D image individual cross-bridges in situ and group (classify) similar structures regardless of position within the lattice ultimately replaced OSR.

The application of ET to muscle thin sections solved most of the problems associated with intrinsic disorder in the filament lattice, as described below. In ET, the entire myac layer is reconstructed as a single volume. Subvolume classification and averaging, which was a later development, were subsequently used to separate and group the different structures and arrangements prior to averaging [31]. Determination of the atomic structures of an actin monomer [41], the myosin head [27] and their combination into an atomic model of F-actin decorated with rigor myosin heads [42] initiated the process of atomic modeling of various states of the cross-bridge cycle within 3D images of muscle [32,43]. 

Atomic model building proved to be key for understanding the structure of myosin cross-bridges in situ. Although many crystal structures of myosin S1 or motor domains (MD) have been produced over the years, only one actin-bound state is characterized at high resolution, nucleotide-free acto-S1. Weak-binding actomyosin states are literally uncharacterized structurally. Weak actomyosin interactions occur frequently in muscle either with nucleotide analogs or as kinetic steps in active contractions. In many instances the myosin head crystal structure and/or its orientation on actin must be modified to fit the reconstruction. The necessity of these modifications is, in fact, one of the more significant observations to emerge from 3D imaging actomyosin in situ.

## 3. Early 3D Reconstruction Work

### 3.1. How Well-Ordered is the Insect Flight Muscle Lattice?

The *Lethocerus* flight muscle lattice, while well-ordered for a muscle, has certain kinds of intrinsic disorder that blur fine details in spatially averaged reconstructions. One important disorder is the mismatch between helical spacings of the thick and thin filaments. The 28/13 actin filament symmetry has an axial repeat period of 77.3 nm. The thick filament axial repeat is 116 nm. The smallest common period is 232 nm, of which there are about four full repeats in a half sarcomere, but four repeats is too few to be useful for averaging purposes. The half pitch of the thin filament is 38.7 nm, which is 1/3 of the thick filament period. Using the axial repeat of 116 nm enabled the myosin and actin lattices to be reconstructed in the same operation. The second disorder is intrinsic to the arrangement of the thin filaments, which have a random 180° azimuthal rotation [4]. The azimuthal disorder generally prevents any actin subunits from being resolved in a spatially averaged reconstruction because two orientations offset axially by the actin subunit spacing of 2.75 nm are averaged. Despite this, an important aspect of 3D images obtained by spatial averages was the ability to validate the observations using EMs of myac and actin layers. 

### 3.2. Rigor Muscle Spatial Average Reconstructions

The earliest 3D reconstruction work on muscle was confined to the rigor state. EM of thin sections had established the double chevron motif of the rigor *Lethocerus* flight muscle [1,5]. The double chevron (Figure 1C) consisted of a large, angled cross-bridge pair towards the M-line, the lead chevron, separated from a smaller cross-bridge pair towards the Z-disk, the rear chevron, by a gap, the intra-doublet gap, that likely corresponded to a single actin subunit on each long pitch actin strand. Because only certain actin subunits in each half period were decorated by myosin heads, the region actually labeled was first dubbed the “target segment” [12], which later evolved into the “target zone” [22]. Of the 14-actin subunits in each crossover, eight constituted the target zone of rigor muscle, four symmetrically placed on each long pitch strand. Later work as described below, showed that the two-actin subunits of the intra-doublet gap were rarely labeled by myosin heads regardless of the presence or absence of bound nucleotide. The eight-actin subunit target zone was later separated into a pair of lead- and a pair of rear-bridge target zones. Subsequent work described below showed that once the nucleotide was added, stereospecific labeling by myosin heads to rear-bridge target zone actins disappeared, replaced by the disordered myosin head contacts to actin and/or Tn. Conversely, myosin head labeling of lead-bridge target zones retained some degree of order, but with greater heterogeneity. In the following discussion, unless specified otherwise, the term “target zone” will refer to the region where rigor lead bridges bind. 

The first 3D image of a rigor flight muscle myac layer using a dual axis tilt series and reconstructed as a 2D array, showed the double chevron motif with a large, dense cross-bridge oriented near the classic, 55° rigor angle and another more diffuse cross-bridge positioned closer to the Z-disk at a 78° angle [22]. At the time, this was the first example of myosin heads attached to actin at other than the “rigor” angle. Actin subunits were not resolved due to the thin filament intrinsic disorder, nor was the troponin complex identified. The troponin position was later identified by gold-Fab labeling which placed it near or at the rear chevron position [44]. 

**Figure 3:**
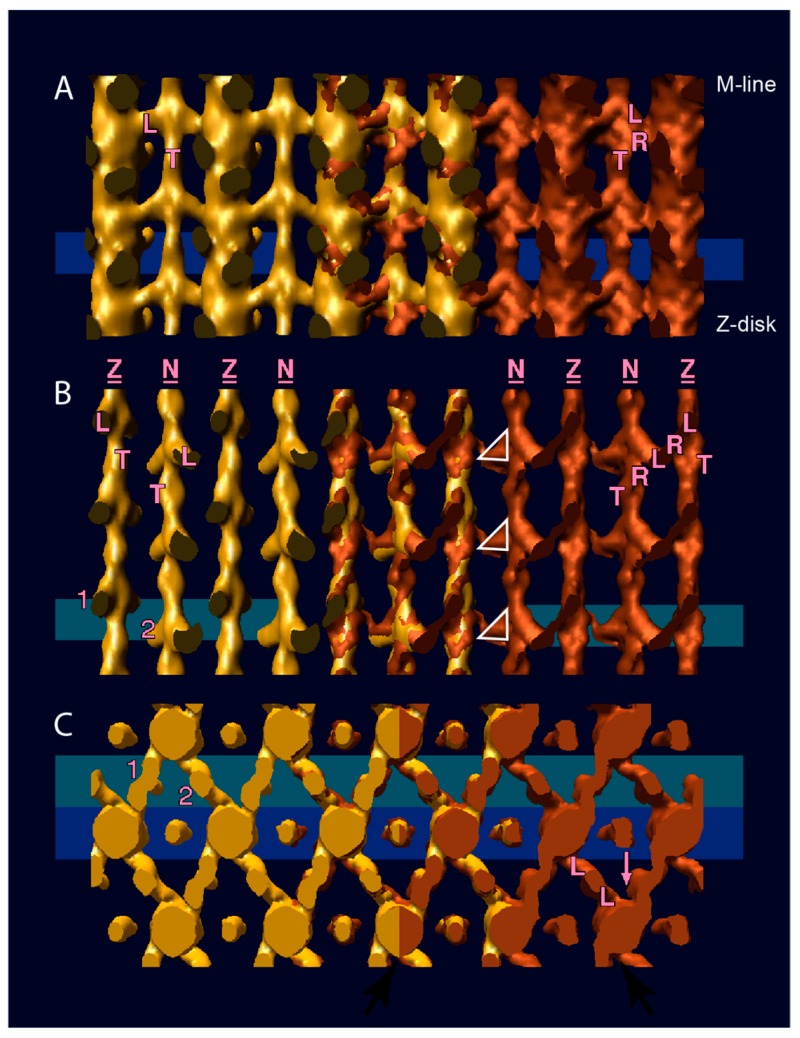
Surface views of oblique section image reconstructions (OSRs) from IHM treated with adenylyl-imidodiphosphate (AMPPNP), a non-hydrolysable analog of ATP at 23 °C (on the left in gold color) and in rigor (on the right in copper color), superposition of rigor and AMPPNP in the middle. (**A**) Myac layers; (**B**) actin layers; (**C**) transverse view of unflared-X layer (AMPPNP on the left) and flared-X layer (rigor on the right). The region in the myac layer of (**A**) and the region of the actin layer of (**B**) contained in the transverse view in (**C**) are marked by the Wedgewood blue and teal green backgrounds respectively. L, Lead bridge; R, rear bridge; T, troponin. Triangles in (**B**) highlight the triangular shape of rigor lead bridges. Lead bridges of rigor and AMPPNP do not coincide exactly in shape so that in the superimposed region in the center, extra mass in rigor shows as copper color. The troponin density is more pronounced in rigor, possibly because the thin filaments are better ordered. In (**B**) the cross-bridge pair that produces the two arms of the unflared-X are labeled 1 and 2. These same cross-bridges are similarly labeled in (**C**). In (**C**) the rigor rear bridge is located at the far surface of the flared-X layer (denoted by the arrow). The extra density provided by the rigor rear bridge results in the close proximity of the vertex arms in projections of flared-X. Reprinted from [28] with permission from Elsevier.

This reconstruction was subsequently improved by the addition of data from thick transverse and longitudinal sections [25], which provided two views mutually orthogonal to the in-plane projection (the 0° tilt). The additional data from thick sections had the effect of better defining the size and shape of the cross-bridges and removing an apparent separation between the two long pitch strands of the thin filament. The lead bridge took on a triangular shape with one edge of the triangle positioned on the thin filament; the second edge on the M-line side gave the lead bridge a strongly tilted appearance. The third edge on the Z-disk side was oriented near perpendicular to the filament axis and one vertex of the triangle, the head-rod junction, was positioned on the thick filament surface (e.g., triangles in Figure 3B). Although the two heads of the lead cross-bridge are not resolved, the shape is suggestive of one head, the leading, or M-line side head having a lever arm tilted at the classic rigor angle, and the trailing, or Z-line head, being less tilted toward the rigor and more perpendicular to the filament axis. 

The OSR recapitulated these findings using a reconstruction procedure that suffers no missing pyramid such as occurs in a dual-axis tilt series, while reconstructing the protein density within a complete unit cell [40]. The OSR is also a spatial averaging technique so actin subunits could not be resolved. The resolution remained limited along the filament axis, but the advantages of the OSR outweighed this problem. Generally, the results of these various reconstructions on the rigor muscle indicated that the lead bridge consisted of two heads of a single myosin molecule diverging from a common origin at the head-rod junction and terminating on adjacent actin subunits on the thin filament. The rear bridge was probably a single myosin head. Some rear bridge sites were unlabeled by myosin heads in these reconstructions, an observation confirmed by an examination of original myac layer micrographs which show frequent “single chevrons”, e.g., Figure 1C, [45,46]. That the lead bridges were favored over rear bridges could be accounted for if 100% of the available actin sites for lead bridges, two subunits per target zone, were filled by the two heads of one myosin, whereas the rear bridge density could be accounted for if a single head occupied 75% of the rear bridge sites on each actin long pitch strand [22]. Some rear bridge targets remained unlabeled by myosin heads. Myosin heads not bound to actin were disordered. The distribution predicted that 78% of the myosin heads would be bound to actin in rigor, a value consistent with several independent measurements [47,48,49]. Thus, myosin binding to lead-bridge actin targets appeared favored even in this early work and remained so in subsequent studies.

### 3.3. Spatial Averages of AMPPNP Treated Muscle

Muscle states produced using non-hydrolysable ATP analogs (AMPPNP), referred to as static states as opposed to dynamic states produced with ATP, were a research emphasis in the 1980s–1990s before the development of rapid freezing techniques that could trap active cross-bridges. Besides the rigor flight muscle, the only static states that had been characterized mechanically, by X-ray fiber diffraction and biochemically were those produced by the non-hydrolysable analogue AMPPNP with or without ethylene glycol [50,51,52]. AMPPNP and ethylene glycol act synergistically in both vertebrate and flight muscle fibers. The addition of either causes a drop in tension (a measure of strong actin binding by myosin) with little change in stiffness (a measure of any actin binding, weak or strong, by myosin) but together their effect is enhanced [51]. At glycol concentrations where fiber stiffness drops to that of relaxed muscle at room temperature, stiffness can be restored by the addition of calcium or lowering the temperature to 0 °C [52]. At room temperature in a solution with regulated actin [53] or in vertebrate fibers at 0 °C in the presence of glycol [54], myosin head binding is increased in the absence of calcium. Thus, the calcium dependent status of the thin filament as well as temperature can influence this process. 

Because it changed the structure of rigor flight muscle myofibrils, as observed by both X-ray diffraction as well as EM, AMPPNP was a favorite subject for finding intermediate cross-bridge structures that might be related to changes occurring during muscle contraction. However, the interpretation of AMPPNP effects were generally controversial regarding the binding affinity of attached and detached myosin heads which can be variable between myosin IIs from different species (see [52] for a discussion of how these issues affect flight muscle). Myosin does not cleave AMPPNP so the addition of the ATP analogue does not by itself relax the muscle, nor can the muscle sustain contractions using the analogue. 

The addition of AMPPNP to the rigor muscle produced variable results. The X-ray diffraction indicated a state different from rigor or relaxed, but the EM of fixed, embedded, sectioned and stained fibers generally looked relaxed [55]. The relaxed appearance was shown to be a preparation artifact that could be avoided by careful monitoring by the X-ray fiber diffraction for changes induced by fixation [56,57]. Certain characteristics of the “AMPPNP state” such as a strengthened 10.6 reflection (innermost reflection on the 19.2 nm layer line) in combination with a weakened 10.3 reflection (innermost reflection on the 38.7 nm layer line) must be preserved to retain any signature of AMPPNP addition at 4 °C [46]. The ratio I_10.6_/I_10.3_, which is not intermediate between rigor and relaxed states, proved to be the yardstick whereby preservation of the AMPPNP effect could be judged. When mechanically monitored for changes in tension and stiffness, the aqueous AMPPNP addition reduced tension while retaining high stiffness [52]. The mechanical effects were not easily explained.

Original images as well as optical filtered images of rigor and AMPPNP myac layers showed frequent cross-bridge binding in both states at a point midway between successive dark beads on the thin filament, the Tn complex. Rigor rear bridges bind the thin filament near to or at the Tn complex, but little cross-bridge marking of Tn was seen in AMPPNP [46,52]. The point between troponins where rigor lead bridges bind later proved to be the location of strong binding in active contraction [34,58]. X-ray studies on the *Lethocerus* flight muscle fibers in the active contraction show an I_10.6_/I_10.3_ increase making this feature diagnostic of myosin head binding at this location [59]. 

Micrographs of thin sections through these static states complement the 3D imaging. Thin transverse sections of the rigor flight muscle show a distinct motif dubbed the “flared X” [5,12,45], which comprises four cross-bridges, apparently originating from two opposing sites on the thick filament instead of the four sites dictated by the filament symmetry, and terminating at four of the surrounding six thin filaments. Two of these bridges were lead bridges and two were rear bridges of the double chevron [45]. Atomic model building of rigor lead and rear bridges in several studies indicated that the lever arm of the myosin head must be bent azimuthally to form the flared X, more so for the rear bridge than the lead bridge, suggesting considerable azimuthal flexibility in the myosin lever arm [32,43]. When AMPPNP is added, the four arms of the “X” straighten into an “unflared X” reflecting a change from apparently two toward the four myosin head origins on the thick filament [46,50,52]. This return toward the 4-fold origins of myosin heads from the 2-fold origins in the rigor flared X would now be interpreted as a change in the lever arm azimuth possibly with changes in the position of the MD on the actin, described in more detail below. Original images as well as the 3D reconstructions described below show that rigor-like rear bridges disappear when nucleotide is added and are replaced by much less regular myosin head attachments in the Tn region. Axial changes in the lever arm appear as an enhanced 14.5 nm periodicity indicating a return to the original *axial* myosin head origin. The azimuthal changes in the rigor-like rear bridges, which have almost a 90° azimuthal change in the lever arm position compared to crystal structures of nucleotide free myosin, probably return to a conformation more in keeping with the crystal structures and thus cannot bind actin or the thin filament in the same way they do in rigor. 

The AMPPNP results at 4 °C were interpreted in terms of the myosin head having two domains [46]. Domain 1 contains the actin-binding interface and Domain 2, which is close to the myosin surface, reflects the myosin head origin. Domain 1, which we would now interpret as the MD, reports nucleotide-induced changes in its position on actin. Domain 2, which we would now interpret as the myosin lever arm, would reflect nucleotide-induced changes in the apparent origin at the thick filament surface. The binding of nucleotide to actin-attached heads changes Domain 2 sufficiently to enable it to follow the helical origin of myosin heads, possibly in concert with a change in the position of Domain 1 on actin. The interpretation was compatible with a hypothesis that the myosin head consisted of two domains, one that bound actin tightly and was consequently immobilized, with another domain moving to produce muscle shortening [60]. The first myosin head crystal structure would not be solved for another six years [27] providing details of these two domains. 

At 23 °C in 4 mM EGTA, the tension and 40 Hz stiffness changes of AMPPNP treated myofibrils are complete, but the structural changes are more subtle than those observed at 4 °C [52]. The 10.6 spot in optical transforms of myac layers is strongly indicative of the cross-bridge formation midway between Tn densities, i.e., lead-style bridges. The strong density repeating on a 14.5 nm period is also seen on the thick filament. The density corresponding to rear bridges of rigor is weak or absent in averaged images and best seen in original images where their variability was retained. The unflared X dominates images of 15 nm transverse sections. Because the Fourier transform of images from thin sections of fibers treated with aqueous AMPPNP at 23 °C is richly detailed in thin sections, the OSR was carried out to visualize these structural changes in 3D [28].

The OSR was done using a unique application of the technique, which utilized both transverse and longitudinal oblique sections to determine amplitudes and phases of the sampled Fourier transform [61] and is the most heavily averaged reconstruction done on the *Lethocerus* flight muscle. The reconstruction showed little if any residual density at the position of the rigor rear bridge (Figure 3A,B) and a lead cross-bridge that was less azimuthally bent than was typical of rigor (Figure 3C). The lead bridge appearance agreed with original images as well as a tomographic reconstruction described below. However, the axial angle was less altered from the rigor angle than expected. Comparison of the structure factors (Fourier coefficient amplitudes) determined by the OSR with those determined by the X-ray fiber diffraction of the native tissue was one of the advantages of this reconstruction technique. Precise agreement between the same structure factor was unlikely due to the extreme difference between the specimen preparations, in one case fully hydrated and in the other embedded, sectioned and stained. However, ratios of the same structure factors within the different preparations were informative. The comparison showed the underweight of the 14.5 nm meridional, but retention of many other features typical of the 23 °C AMPPNP state, such as the important increase in the I_10.6_/I_10.3_ intensity ratio. The retention of lead-type cross-bridges foretold the limitation of strong-binding cross-bridges in the actively contracting muscle to the rigor lead-bridge location. The lack of cross-bridges at the rigor rear bridge targets in AMPPNP could explain the large drop in tension, but not the retention of stiffness unless those heads detached by AMPPNP could reattach weakly to the thin filament with less ordering. Atomic models built into 3D images of rigor rear cross-bridges indicated that the attached myosin heads would be highly strained compared with those of lead bridges [43,61] suggesting greater susceptibility to detachment by added nucleotide.

Imaging of the *Lethocerus* flight muscle using averaged reconstruction methods had major limitations, which were always apparent, but not treatable at the time. As long as a structure was regularly arranged, it would appear in the reconstruction, if not, it was either blurred or averaged out and not visible. The intrinsic disorders prevented actin subunits and individual heads of 2-headed myosin attachments to actin from being resolved. Although the OSR had some compensating advantages, e.g., comparison with X-ray fiber diffraction from native fibers, the advantages did not fully compensate the disadvantages. It took ET to bring out the richness of myosin’s interactions with actin in situ. 

## 4. 3D Imaging by Electron Tomography of Static States

ET came into its own as a structural technique when computer-controlled microscopes could collect tilt series automatically [29]. However, the earliest tomogram of the *Lethocerus* flight muscle [30] was obtained from the same dual axis tilt series used for spatial averaging [22] and thus had been manually recorded. Subsequent tomograms of muscle in rigor and treated with AMPPNP also utilized data collected manually [62,63]. The tilt series images were aligned using what is now referred to as marker free alignment, which was unique at the time and later developed into the general alignment tool contained in the program PROTOMO [64,65]. This first “raw” tomogram was virtually impossible to interpret because of various types of noise in the data in the form of stochastic as well as variations in staining and preservation (structural noise). Interpretation required the independent development of subvolume alignment and classification to improve the signal-to-noise ratio and make these features interpretable in some detail [31,66]. Details on methods used for ET and subvolume averaging of the *Lethocerus* muscle can be obtained from several publications [35,67,68,69].

The first atomic model of actomyosin [42], which was obtained by a combination of an actomyosin cryoEM reconstruction with the atomic model of F-actin and the crystal structure of the myosin head, facilitated more detailed interpretation of the tomographic reconstructions. An early development of quantitative atomic model refinement at low resolution was tested using subvolume averages derived from tomograms of rigor muscle [32,43]. In nearly every instance where a cross-bridge could be interpreted as binding actin strongly in situ, this model and more recent versions, required modification of the lever arm to facilitate a fit. Those modifications were greatest for the rigor rear bridge.

**Figure 4:**
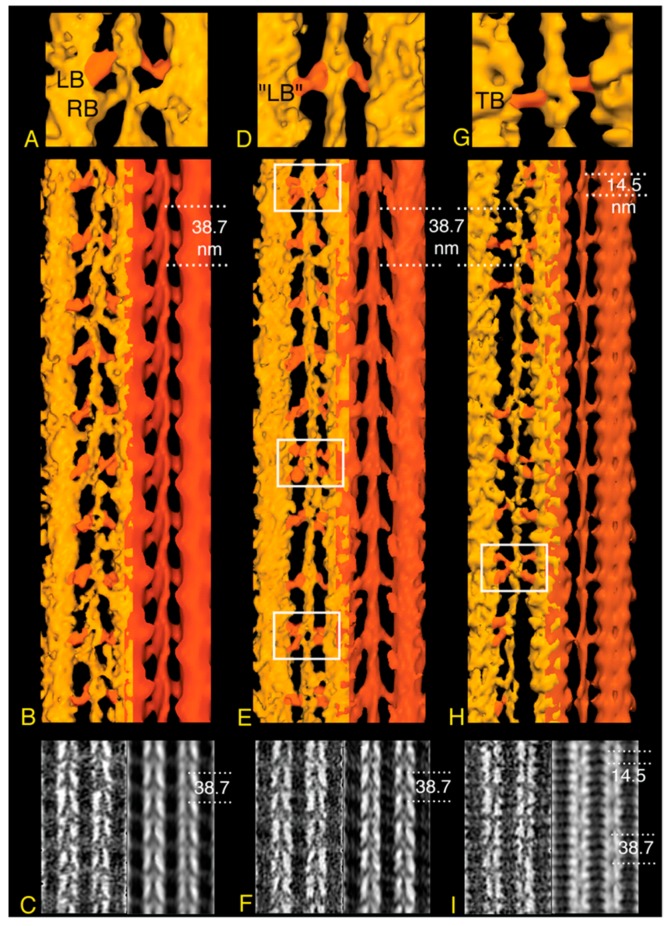
Tomographic image reconstructions of IHM in three equilibrium states: Rigor (**A**–**C**), aqueous AMPPNP at 23 °C (**D**–**F**) (from [62]) and the glycol-AMPPNP at 5 °C (from [63]) (**G**–**I**); (**A**,**D**,**G**) surface renderings of a single 38.7 nm actin period with attached cross-bridges; (**B**,**E**,**H**) surface renderings of a larger area; (**C**,**F**,**I**) projections of a region of the tomograms. The orientation has the Z-disc on the bottom. In (**B**,**E**,**H**), the column-averaged filament is shown in red to the right of the unaveraged filaments (gold) on the left. Target zone cross-bridges are colored red for ease of identification. The comparison clarifies the main differences among the three states; (**A**–**C**) the rigor state shows well-ordered, double chevrons consisting of lead- and rear-bridge pairs every 38.7 nm. There is no 14.5 nm periodicity visible on the myosin filament surface; (**D**–**F**) in aqueous-AMPPNP, the lead bridge motif every 38.7 nm is retained, often appearing single-headed, while rear bridges become disordered. Weak 14.5 nm periodicity can be seen on the myosin filament surface. White boxes outline mask motifs; (**G**–**I**) the glycol-PNP state shows single-headed attached cross-bridges every 38.7 nm, but their size, shape, and attachment angle generally differs from the rigor and AMPPNP lead bridges. A mask motif is outlined by the white box. The myosin filament surface reveals a strong 14.5 nm repeat of cross-bridge shelves. ©1997 Schmitz et al. Originally published in Journal of Cell Biology. https://doi.org/10.1083/jcb.139.3.695.

Several modifications of the rigor *Lethocerus* flight muscle were imaged by ET including the rigor muscle swollen in low ionic strength buffer, which revealed the length of the proximal S2 domain that functions as a tether for the myosin heads [70]. Another study applied a stretch to the rigor muscle fibers followed by rapid freezing-freeze substitution to reveal elastic distortions in the myosin lever arm [33]. The static states of the *Lethocerus* flight muscle imaged using ET included rigor and aqueous AMPPNP at 23 °C [62] as well as the mixture of AMPPNP and ethylene glycol obtained at 5 °C, known as the “cold, glycol stiff state” [63]. The tomograms of the rigor muscle recapitulated interpretations made earlier based on spatial averages (Figure 4A–C). Lead chevrons appeared in every 38.7 nm thin filament repeat midway between the troponin densities. Rear bridges were less regular and generally smaller when they were visible with their lever arms at variable angles. When averaged, the rear bridges became a diffuse density on the Z-ward side of the lead chevron that in isodensity display appeared smaller and less angled than lead bridges. Multivariate data analysis of the repeating motifs, a process that can identify similar structures within a variable ensemble, had not been developed at this time. Instead, a novel averaging method dubbed column averaging was used. In column averaging successive 116 nm periods are averaged, but only along the axial direction; there is no lateral averaging between filaments. Column averages had less signal-to-noise improvement than spatial averages, but retained more of the cross-bridge variability.

Tomograms of fibers treated with aqueous AMPPNP at 23 °C (Figure 4D–F) generally showed retention of lead cross-bridges, but they were more variable in both size and axial angle [62] consistent with original micrographs and optically filtered images [46,50,52]. In column averages, they appeared as a repeating pattern of apparently 2-headed and 1-headed lead bridges. Rear type bridges disappeared even in the column averages, but were visible in the raw tomogram. Thus, the tension and stiffness effects of aqueous AMPPNP could be explained by the detachment of strong-binding rigor rear bridges that reattach weakly to the thin filament with disorder. A new motif, dubbed the “mask motif” (Figure 4E) because of its resemblance to a harlequin mask, appeared for the first time [62]. The mask motif consisted of a “lead-type” bridge in combination with a myosin head attaching near it, but coming from the neighboring M-ward crown. In rigor myac layers, paired rear bridges always bound the thin filament symmetrically about the filament axis and approached the thin filament from symmetric azimuths on the thick filament. In AMPPNP, this was not true as myosin heads contacting the rear bridge sites on the thin filament approached from nearly any direction on the thick filament, either front or back side. Their disordered appearance was consistent with nonspecific, weak attachments. Thus, cross-bridges in AMPPNP interacted with actin in ways different from rigor attachments.

The availability of a rigor actomyosin atomic model facilitated a more detailed interpretation of the rigor tomograms. When placed in the reconstruction, MDs of the two actin-attached heads fit within the reconstruction envelope, but the lever arms of both heads would have to be adjusted azimuthally and axially if the two heads were to come together at a common origin, the head-rod junction [32,62], and still fit in the density. The lever arm of rigor rear bridges, on the other hand, required nearly a 90° azimuthal bend as well as axial adjustments to fit in the envelope [32,62]. The fittings of the rigor rear bridge based on nucleotide-free acto-S1 demonstrate that in situ large lever arm deformations in the myosin head are possible when attaching actin in a state of high affinity. Thus, the tomograms provided a direct observation consistent with the proposal that strain-enhanced detachment of cross-bridges explained the effects of AMPPNP in vertebrate preparations [71,72]; in flight muscle rear bridges were the most strained. The preferential retention of ‘‘lead’’ bridges in AMPPNP implied an arrangement of myosin head conformation and thick filament origins that favored two-headed binding and less cross-bridge strain at their actin targets. However, the frequent appearance of single-headed “lead” bridge in AMPPNP at 23 °C indicated that the reduction in actin affinity was sufficient to prevent attachment by second heads in some instances. As in the rigor tomogram, there was no clear indication where heads not attached to the thin filament were located. 

Although in AMPPNP rigor rear bridges detached, they were reattaching to actin in ways not previously seen, but clearly different from the very specific attachment to actin found for strong binding myosin heads in rigor [62]. In most instances, there was no clear way the actin attachment could convert to the stereospecific attachment of rigor except in one instance, the mask motif, for which the attachment azimuth of the M-ward head to actin was reasonably similar to the position of the Z-ward actin-bound “lead” bridge head (Figure 4E,H). The arrangement was suggestive of a relay mechanism in which one target zone is transferred from one “crown” to the next crown on the M-ward side during sarcomere shortening. Subsequent information as described below indicates that M-ward bridges of mask motifs in AMPPNP must be very different from similar structures seen in the active muscle.

Adding ethylene glycol to the AMPPNP solution drove the muscle from rigor even more towards a relaxed appearance, while retaining stiffness nearly equal to that of rigor [50]. Up to a “critical glycol concentration” of about 30%, flight muscle fibers were mechanically as stiff as rigor fibers, but would not sustain applied tension. Tomograms of this glycol-stiff state in flight muscle fibers showed a significant structural change in the actin-bound heads (Figure 4G–I). Whereas rigor and AMPPNP treated fibers at 23°C displayed target-zone attachments of a similar structure, target zone attachments in the glycol-stiff state were different [63]. The actin azimuths for target zone cross-bridges in rigor and AMPPNP had a narrow distribution, the distribution of glycol stiff target zone cross-bridges was much broader, though still centered at the same average azimuth. Axially, rigor and AMPPNP target zone cross-bridges tended toward a 45° axial angle, glycol-stiff cross-bridges averaged closer to 90°, but with a broader distribution. Despite the large change in their structure, glycol-stiff, lead-bridge attachments retained the azimuthally symmetrical attachment to the actin targets characteristic of some specificity in their interaction with actin. Mask motifs could still be observed in the glycol-stiff state (Figure 4H). Outside of the target zone, azimuthally, cross-bridges attached to just about every accessible surface of the thin filament. Thick filaments were marked by a strong 14.5 nm axial period [63]. 

The results from ET studies of rigor and these static states suggested that, as the affinity of the myosin head attaching to actin increased, the MD could position itself on actin independent of the lever arm, which must accommodate the thick filament origin as well as changes in the MD on actin [63]. This interpretation differed from other models that placed the MD on actin in all strong binding states in a single, stereospecific orientation with the lever arm moving only axially to produce filament sliding [42]. It also differed from models in which the entire myosin head rotated on actin during force production [2,73]. 

### Impact of New Information 

Several results published since the work described above for static, nucleotide-bound states of the *Lethocerus* flight muscle appeared to bear on their interpretation. These results include X-ray crystal structures of myosin II intermediate states obtained from MD constructs of *Dictyostelium discoideum*, high-resolution structures of actomyosin by cryoEM, and the structure of relaxed striated muscle thick filaments from several species.

The crystal structure of the *D. discoideum* myosin II MD in the presence of AMPPNP proved to be very similar to structures of the same construct in the presence of MgATPγS [74], MgADP•BeFx [75] and pyrophosphate [76]. These analogs produce structures that we would now classify as post-rigor, myosin•ATP-like conformations in which the lever arm is oriented down, characteristic of the end of the power stroke and the actin binding cleft, also called the 50 kDa cleft, is open, characteristic of a weak-binding conformation [77]. Numerous crystal structures of complete myosin heads obtained from molluscan sources have similar features [78,79,80,81]. How do these results impact the tomographic reconstructions of the *Lethocerus* flight muscle in the presence of AMPPNP with or without ethylene glycol?

1. Assuming the AMPPNP addition to *Lethocerus* myosin also produces a post-rigor conformation, the head would have its lever arm down and its actin binding cleft open. Such a conformation would explain why lead bridges in aqueous AMPPNP at 23 °C resembled rigor lead bridges when viewed in longitudinal sections. That sometimes they appeared to be single headed would reflect an effect of actin subunit azimuth in relationship to the lead bridge origin on the filament backbone. The comparatively weak actin-binding affinity of myosin•AMPPNP may not sustain large changes in the lever arm required to bind actin with the second head in some instances when one head is already bound. An open actin binding cleft with its correspondingly lower actin-binding affinity when AMPPNP is bound would explain the systematic loss of the highly distorted rigor rear bridges. On the other hand, with the lever arm down, the M-ward cross-bridge of mask motifs and other thin filament attachments outside of the target zone must differ from similar structures identified subsequently from the active muscle described below. The mask motifs of the active muscle have a lever-arm up conformation characteristic of the myosin II transition state, not a lever arm down conformation, and they seem to be contacting TM rather than actin in the active muscle. Conceivably, they might be held near the thin filament by a structure other than the myosin MD, perhaps a long N-terminal extension of the RLC of myosin, in which case, the up or down state of the lever arm would be relatively unimportant. A connection between the RLC and actin seems to occur in the *Drosophila* flight muscle [82,83]. The *Lethocerus* flight muscle RLC has a similar N-terminal extension (Belinda Bullard, unpublished).
2. In aqueous AMPPNP, there is a stronger periodicity along the 14.5 nm repeat of myosin. Ordering of the RLC at the head rod junction probably contributes too little mass to account for such an effect. Increasing ethylene glycol up to the critical glycol concentration of ~30% moves the appearance even more toward that of the relaxed muscle while retaining rigor stiffness [52]. However, unless ethylene glycol can drive myosin•AMPPNP toward the transition state by cleaving the nucleotide, an unlikely though unproven possibility, the thick filament structure cannot fully relax by forming the IHM. So why does it take on a relaxed appearance in the glycol-stiff state and ultimately take on the appearance of fully relaxed muscle?

**Figure 5:**
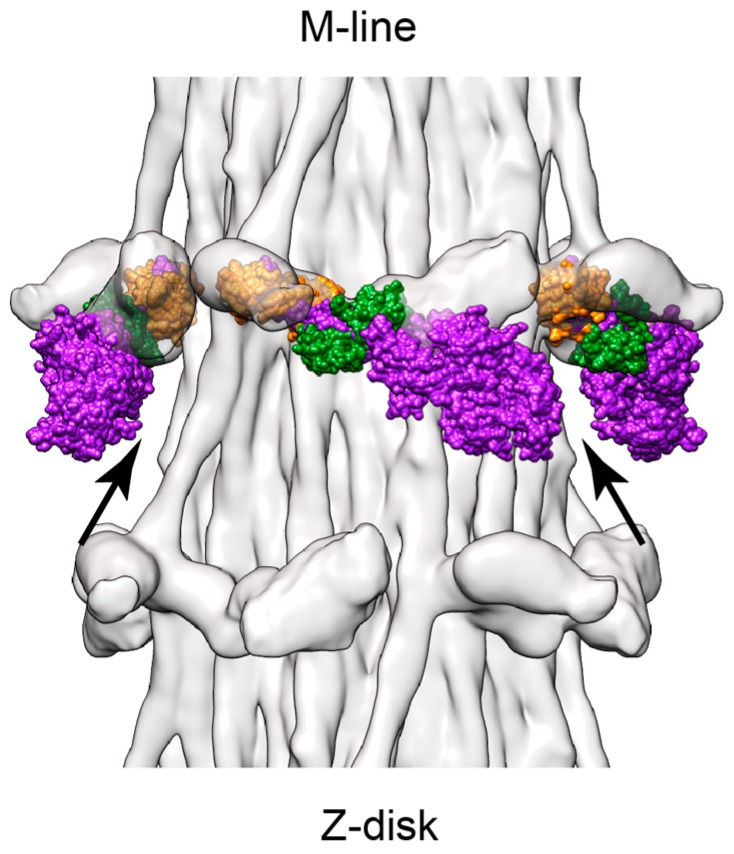
Fitting of a post rigor myosin head conformation into the relaxed *Lethocerus* thick filament. The atomic model of *Doryteuthis pealeii* (squid), PDB 3i5f, a post-rigor conformation [82] fit using only the RLC portion of the lever arm onto the free head of atomic model of [6]. Arrows point to the space between the free head and the myosin rods in the backbone; there are no clashes between the MD and the backbone. The related rigor-like structure, PDB 3i5g, fits equally well. The degree of clash observed with other post rigor crystal structures having complete lever arms, e.g., 2mys and 1dlf, fit using the same criterion is variable. Coloring scheme has the RLC (orange), ELC (green) and heavy chain (purple). Note that the blocked head density and atomic model have been removed for clarity.

We hypothesize that ethylene glycol in increasing amounts progressively weakens the affinity of myosin•AMPPNP for actin, leading first to dynamic, single-headed weak attachments. When the presumptive free head of a myosin molecule becomes detached from actin, the equilibrium would shift with increasing glycol concentrations so that free heads that are detached from actin would bind the filament backbone more or less as “free” heads in relaxed thick filaments. The free-head backbone attachment in the relaxed *Lethocerus* thick filaments is primarily through the RLC [6]; the position of the MD may be less important for this backbone attachment unless it clashes. Molluscan post-rigor myosin head structures, when aligned using the RLC and its bound heavy chain segment to the interacting heads motif of relaxed *Lethocerus* thick filaments, fit with no or only minor clashes between the MD and the thick filament backbone (Figure 5). If the free head attaches the filament backbone, the “blocked head” would have restricted movement due to the free head pinning the S1-S2 junction onto the backbone; the essentially “free” blocked head must pivot about its junction with S2 rather than the location where S2 leaves the thick filament backbone ~11 nm away. The pyrophosphate addition to rigor the *Lethocerus* flight muscle also produces a structure similar to that of AMPPNP plus ethylene glycol with an enhanced 14.5 nm meridional periodicity characteristic of the relaxed muscle, but without ATP cleavage to reposition the lever arm to the transition state [84].

## 5. Electron Tomography of Frozen Active Muscle

The first studies attempting to visualize active cross-bridges in the rapidly frozen, freeze substituted muscle were done on the vertebrate striated muscle [24,85,86,87,88], but none of these efforts led to 3D imaging and atomic model building. The first ET studies of the rapidly frozen *Lethocerus* muscle were done on stretched rigor fibers [33]. Subsequently, the rapid freezing of active *Lethocerus* muscle with simultaneous tension monitoring in the presence of ATP and Ca^2+^ was undertaken. Myosin cross-bridges in the active muscle act as independent force generators so that all steps in the ATPase cycle are present, though not in equal proportion. The active muscle when rapidly frozen provides a snapshot in time of the dynamics of cross-bridge interaction with the thin filament and ATP. The specimen preparation methods used for frozen active flight muscle fibers have been described [35] and so will not be elaborated on here except to say that fibers (muscle cells) from the glycerinated muscle are mounted on a tension transducer, tested for activation and relaxation and then smashed into a liquid-helium-cooled copper block while simultaneously measuring tension. The frozen muscle tissue is then freeze-substituted, embedded, sectioned and stained for ET data collection. The tilt series were initially single-axis, but later became dual-axis. 

The efforts on the *Lethocerus* muscle started with what was then described as a state of High Static Tension, which would now be interpreted as an isometric contraction (iso-HST). At the time, the flight muscle, which operates in stretch activation mode during flight, was deemed incapable of an isometric contraction. Subsequent work showed that an isometric contraction could be generated at pCa < 4.5 [89]; at lower calcium concentrations, stretch activated contractions occurred.

The first tomograms of the active *Lethocerus* flight muscle used the column averaging method described above in which individual filament averages were produced with no averaging between filaments [58]. This process produced a richer population of cross-bridge conformations than could be obtained using spatial averages. In column averages, all of the cross-bridges were bound to the region midway between Tn complexes, which is the same location where rigor lead bridges bind; the raw tomograms also showed cross-bridges at other locations along the thin filament. That most of the cross-bridges in active contraction occurred in the rigor, lead-bridge target zone was presaged by results with AMPPNP described above as well as by X-ray diffraction that showed, in active contraction of *Lethocerus* fibers, the 10.12 reflection on the inner part of the 19.2 nm layer line (the 10.6 reflection above) was enhanced [59]. However, these target zone cross-bridges were far more heterogeneous than the structures observed in rigor or AMPPNP.

The column averages of target zone bridges in the raw iso-HST tomogram clustered into three lever arm orientations, 125° (pre-power stroke), 105°, and 70° (rigor-like). Each target zone cross-bridge was consistent in size with a single myosin head. The mask motifs similar to those of aqueous AMPPNP were present in quantity.

Atomic model building at the time was limited by the number of myosin head structures that had been determined with the complete lever arm present (one) [27]; most structures were of the MD alone, sometimes with only the ELC. In the year 2000, the transition state structure of scallop myosin II appeared [78]; all the S1 crystal structures obtained from the molluscan muscle have a complete light chain-binding domain and thus complete lever arms. In lieu of a transition state crystal structure, the iso-HST cross-bridges were modeled by rebuilding the post-rigor skeletal S1 structure using G770 as the pivot point, comparing the result to a model transition state structure [90]. However, even with that degree of added flexibility in the skeletal atomic structure, reasonable fits could not be obtained without also moving the MD away from its position in rigor acto-S1. Large azimuthal changes in the lever arm position were necessary in the majority of cases. Taken together, the models suggested a 2-stage power stroke. In stage-1, the axial orientations of the motor and light chain domains change together; in stage-2 the MD does not change its orientation and only the lever arm changes its axial angle. The total interaction distance amounted to 13 nm of which 10 nm produced positive work and 3 nm negative work. The axial lever arm change with the MD fixed on actin, i.e., the strong force producing stage, amounted to 5 nm. 

## 6. Myosin Head Structures in Frozen Active Muscle

**Figure 6:**
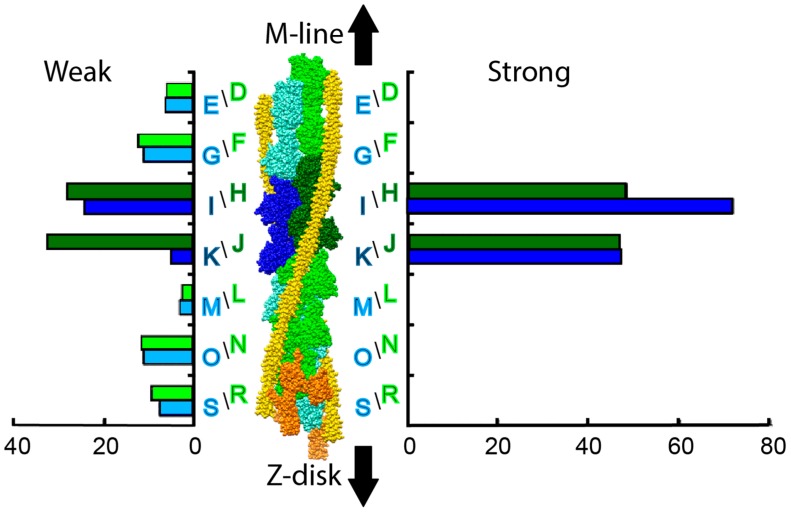
Myosin head binding to specific actin subunits on the 38.7 nm thin filament repeat in the *Lethocerus* flight muscle undergoing an isometric contraction. On the left weak attachments are shown; on the right are strong attachments. Actin subunits on the two long pitch strands are colored green and blue. The two target-zone actin subunits are in darker shades. Actin subunit designations correspond to the chain names in the coordinate files deposited in the Protein Data Bank, PDB–2w49. From [34].

Developments in ET produced methods to align and classify the heterogeneous individual motifs (38.7 nm segments of the thin filament) from muscle tomograms [31,66,68], which improved the resolution 2.5x over column averaging. For the first time, individual actin subunits and myosin heads could be resolved in class averages facilitating the detailed study of myosin head conformations in the fast frozen, active muscle. Three states of active muscle were examined by ET, isometric contraction (iso-HST) and isometric contraction following a quick stretch (str-HST) or a quick release (rls-HST). The ability to fit the thin filament atomic model independently of the cross-bridges combined with the availability of crystal structures of rigor, post rigor and transition states of S1 produced improved atomic models. The new methodology, not only provided resolution to distinguish individual myosin heads and actin subunits, but was sufficient to develop a criterion for differentiating weak from strong myosin attachments and for quantitating the relative numbers of the different structures [68]. Weak actin attachments were differentiated from strong actin attachments by whether or not a MD in the well-known strong binding position fit the density. If it fit, the actin attachment was strong; if not, and the MD of myosin had to be moved to fit the density, the actin attachment was weak. The lever arms invariably required either axial or azimuthal changes, or both, as previously required in the earlier atomic models of rigor and AMPPNP. Once a thin filament attachment in a class average was determined to be weak or strong, the number of class members comprising that class average could be used as a measure of the number of occurrences of that structure on each of the 14 actin subunits in the 38.7 nm axial repeat (Figure 6). Quantification of each different actin-bound state of myosin on an individual actin subunit of the thin filament motif has not been duplicated in any other system. 

Surprisingly perhaps, in iso-HST, cross-bridging density attributable to myosin heads was found on all 14-actin subunits in the 38.7 nm thin filament repeat (Figure 6). Strong-binding attachments were found on only the four-actin subunits exactly midway between successive Tn complexes, i.e., the target zone of lead bridges in rigor and AMPPNP. Weak attachments were found everywhere else, including within the target zone of strong-binding cross-bridges. Very few myosin head attachments were found on the two-actin subunits on the Z-disk side of the target zone (M and L in Figure 6); these were the same actin subunits comprising the “intra-doublet gap” of rigor flight muscle. These subunits apparently present a very unfavorable actin azimuth for *any* myosin head attachment. 

**Figure 7:**
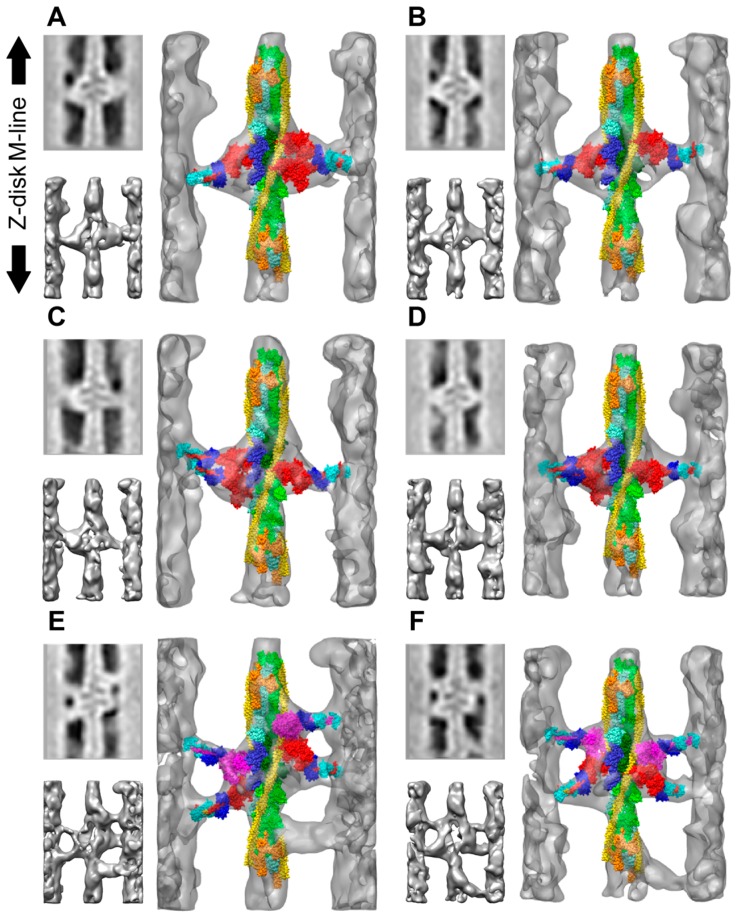
Class averages and quasi-atomic models of a selection of class averages from the *Lethocerus* flight muscle in an isometric contraction. Small panels to the left are the central section (top) and an opaque isodensity surface view (bottom) of the larger panel. In the translucent larger panel, actin long pitch strands are cyan and green with the target-zone actins in darker shades, TM is yellow and Tn orange. Heavy chains of strongly bound myosin heads are colored red, weak binding myosin heads magenta, ELC dark blue and RLC light blue. (**A**) Single headed cross-bridge on the left and a 2-headed, strong-binding cross-bridge on the right; (**B**) a pair of 1-headed, strong-binding cross-bridges on actin subunits H and I; (**C**,**D**) a strongly bound 2-headed cross-bridge on the left and a strongly bound 1-headed cross-bridge on the right. (**E**,**F**) are mask motifs with Tn-bridges. Tn-bridges have not been fit with a myosin head; (**E**) the right, M-ward side, cross-bridge is a weak binding Type 2 bridge outside of the target zone contacting TM near actin subunit F, while on the left is a Type 1 weak-binding cross-bridge within the target zone on actin subunit I. (**F**) On the M-ward left side is a Type 2 weak-binding, cross-bridge contacting TM outside the target zone near actin subunit G, while the Type 1, weak-binding cross-bridge on the right is contacting target-zone actin subunit H. From [34].

Weak-binding attachments were grouped into three types; 1, 2 and Tn bridges. Type 1 weak attachments were restricted to the target zone and thus interpreted as precursors of strong-binding cross-bridges, or pre-power stroke cross-bridges (Figure 7E,F). Type 2 weak attachments were found both in the target zone and just M-ward of the target zone, often as part of the mask-motif structure (Figure 7E,F). Their MD contact with the thin filament was generally through TM and not actin. Hence they were later dubbed TM bridges. Their means of actin attachment was not clear, perhaps being through a long N-terminal extension of the RLC as seems to occur in the *Drosophila* flight muscle [82,83]. They had no clear path to strong binding as long as they were positioned M-ward of the target zone. To convert to strong binding required a change in the azimuth of the actin subunit, which would occur if the target zone were “relayed” M-ward during sarcomere shortening. 

Tn bridges formed a highly heterogeneous set of attachments on either Tn or the actin subunits in the same location (Figure 7E,F). They approach this region of the thin filament from a wide variety of thick filament origins as did cross-bridges in the same axial position when AMPPNP was present. Their contacts on the thin filament are at least as variable as those found in other non-target zone locations. Because of their heterogeneity, they were not investigated further. However, they may play a yet-to-be-determined role in stretch activation.

**Figure 8:**
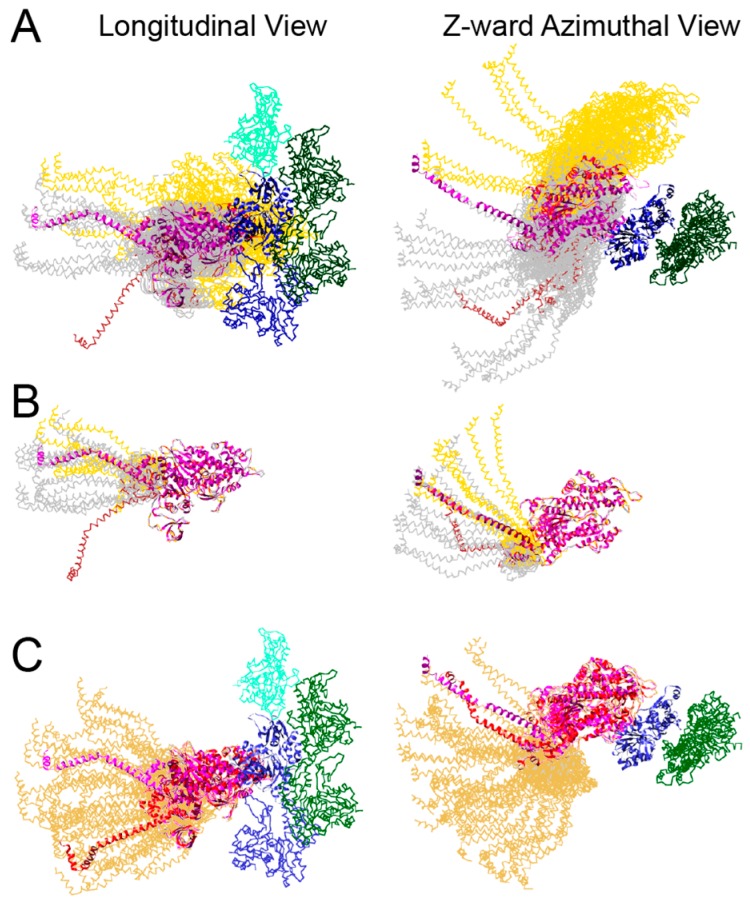
Range of lever arm positions for strongly and weakly bound cross-bridges in isometrically contracting *Lethocerus* flight muscle. Ribbon diagrams of actin subunits are colored blue and green. Ribbon diagrams are shown for only the heavy chains of myosin. (**A**) All weak binding cross-bridges superimposed on actin subunit I (see Figure 6). This view illustrates the variations in MD position when referred to a single actin subunit. Chain traces of Type 1 bridges are shown in gray and Type 2 bridges in yellow. The single post-rigor conformation found is colored light brown. Note the relatively small axial dispersion of the Type 1 MDs compared to the broad dispersion of the Type 2 MDs; (**B**) all weak-binding cross-bridges aligned on the scallop transition state MD to illustrate lever arm variations compared with the starting scallop S1 structure. Coloring scheme is the same as for panel A; (**C**) rebuilt models of strong-binding cross-bridges are colored (gold) superimposed on both starting myosin head structures (red and magenta) as docked onto actin in the strong binding configuration. Adapted from [34].

The positions of Type 1 weak binding myosin heads showed slightly variable axial positions and orientations, but highly variable azimuthal positions, biased almost exclusively to the anticlockwise direction from the strong binding position relative to the thin filament center (Figure 8A). The alterations to the lever arm relative to the MD of all weak binding bridges (Figure 8B) were much smaller than those for strong-binding cross-bridges (Figure 8C). The small axial variations in the MD of pre-powerstroke cross-bridges were distributed on both sides of the strong binding MD position and thus did not indicate a concerted motion that could contribute to the power stroke. Thus, the pre-powerstroke attachments suggested that the weak to strong transition involved mostly the *azimuthal* movement of the MD across actin subdomain 1 and towards TM in a clockwise direction before actin binding cleft closure. The changes needed to fit the lever arm of pre-powerstroke cross-bridges were smaller than the azimuthal changes, but biased in the anticlockwise direction relative to the starting crystal structure. 

Strong-binding cross-bridges consisted of both single- and double-headed actin attachments and had *axial* lever arm orientations that covered a range of 12.9 nm consistent with measurements of the lever arm swing obtained by other methods [91] as well as in column averages described above (Figure 7A–D). Axial changes in the MD as previously interpreted for strong-binding attachments [58] were visible only on Type 1 weak-binding cross-bridges because the criterion for identifying strong-binding bridges precluded it. However, for nearly all strong-binding attachments, the lever arm azimuths had a variable and very different distribution than predicted by the crystal structures, but covering the same 90° change as required for the rear bridges of rigor flight muscle. Based on the crystal structures available to Wu et al., the junction with S2 for strongly bound myosin heads within the target zone, was expected to be on the clockwise side of the inter-filament axis when looking Z-wards, using the thin filament as the center of reference (see Figure 6 of [34]). The overwhelming majority of in situ cross-bridges originated on the anticlockwise side giving the strong-binding cross-bridges a straightened appearance relative to the two crystal structures used as references. This observation was presaged by the earlier work on flight muscle in rigor, particularly the rear bridges of rigor muscle and the lead bridges of aqueous AMPPNP.

Comparison of the chicken skeletal myosin (PDB–2MYS) with the scallop transition state structure (PDB–1DFL) when both are placed on actin in the strong-binding orientation, shows that azimuthally, their lever arms superimpose (Figure 8C). Wu et al. explored several ways that pre-powerstroke myosin heads can position themselves on actin preceding strong binding to produce the appearance of strong-binding bridges of contracting muscle. These models differed in whether the S2 domain or the myosin lever arm were compliant. In one model, myosin heads are non-compliant and must find an appropriate actin subunit by rapidly attaching and detaching until they contact an actin subunit in an orientation that facilitates strong binding through closure of the actin binding cleft. If the heads are assumed to be non-compliant, their ability to find an appropriate actin subunit depends on flexibility of the S2 whose origin must be on the clockwise side of the inter-filament axis. To make the cross-bridge origin appear as if it comes from the anticlockwise side, as observed, requires an azimuthal swing of the lever arm during the power stroke. 

The other model involved an azimuthal movement of the MD across Subdomain 1 of actin as described above. In this case, the myosin origin was anticlockwise of the inter-filament axis. During the MD’s movement across Subdomain 1, either the lever arm, the S2 domain or both are bent azimuthally. When the power stroke ensues, the S2 aligns with the filament axis, further bending the lever arm azimuthally if necessary. An azimuthal component of the power stroke is not necessary to position the S1–S2 junction in the observed region of the thick filament, i.e., anticlockwise of the inter-filament axis. An axial force transmitted through S2 would be sufficient, provided the myosin head originated from the positions observed in iso-HST.

Studies of the rigor *Lethocerus* flight muscle have characterized the physical dimensions of the S2 as a tether of myosin heads better than for any other striated muscle. The ET of rigor fibers swollen in low ionic strength buffer pulled the S2 tether free of the filament backbone, but revealed only 11 nm of S2 [70]. If the ionic strength was lowered even further, whole ribbons of myosin rods (referred to as subfilaments at the time, but now known to be ribbons) were pulled free of the filament backbone. The 11 nm length of the S2 tether was later confirmed by the high resolution structure of the relaxed thick filament [6]. The length of the S2 that functions as a tether for active myosin heads searching for actin subunits and its consequences for muscle contraction have not been examined in detail, even in *Lethocerus* where the structure is well defined. 

The axial lever arm changes were within expectations, but the implied thick filament origins and the azimuthal variations were novel, implying an aspect of the power stroke not considered in models current at the time or since. Because rigor lead bridges bind to the same target zone as strong-binding cross-bridges in active contraction, their origins could be investigated from transverse sections of rigor fibers swollen in low ionic strength buffer. Arakelian et al. [92] computed tomograms from 80 nm thick transverse sections of swollen rigor fibers and then sectioned the tomograms into thin slices from which the S2 origins could be determined. The distribution of azimuths was roughly Gaussian with a mean of −26° ± 9°, i.e., clockwise of the inter-filament axis, in this case referred to the thick filament as the center of reference. This location would be anticlockwise of the inter-filament axis with the thin filament as the center of reference. The distribution was consistent with the hypothesis that the myosin MD moved azimuthally across its actin-binding site towards the strong binding position, bending either the lever arm or the S2 connection or both in the process. 

The presence of azimuthal movements of myosin across its actin-binding site during the weak to strong transition, implied that a force produced by purely axial lever arm movements might produce a torque on either the thick filament, the thin filament or both. X-ray diffraction of muscle fibers are clear on this issue; changes in the helical pitch of the thin filament are not observed, although the axial repeat is altered by a small amount in response to applied tension [93,94]. In vitro motility assays have observed azimuthal movements of actin filaments produced by myosin, referred to as twirling [95]. If changes in actin filament pitch occur in situ in muscle, they are either (1) local and compensated by changes in the opposite direction in order to maintain the 38.7 nm crossover spacing as suggested in the first 3D reconstruction of rigor flight muscle [22], (2) too small to be detected in an ensemble measurement such as X-ray fiber diffraction, or (3) only affect actin subdomain 1. 

For the thick filament, particularly that from the *Lethocerus* flight muscle, helical changes observed during active contraction are also observed when the relaxed muscle is stretched [96] and thus cannot be attributed to the myosin power stroke. The length changes in *Lethocerus* thick filaments are only 0.05% [96]. An azimuthal component to the power stroke could dissipate any torque produced by an azimuthal movement of S2 as the myosin head moves across an actin subunit. Alternatively, compliance of the lever arm and S2 might dissipate any torque because they are much smaller physically than the filaments themselves.

## 7. Active Muscle Following a Quick Stretch or Release

After characterizing the head distribution in iso-HST, Wu et al. performed a similar analysis on an isometrically contracting muscle subjected to a length transient [97]. Length transients were completed within 2.5 ms and frozen 5.5–6.5 ms after completion of the length change, overall ~9 ms. The elapsed time, though fast for this type of specimen preparation, was too slow to capture the length transient itself, or the immediate structural response. Changes observed in the structure of strong-binding cross-bridges were smaller than expected in the lever arm angle, but larger changes were observed in the distribution of cross-bridge types, both weak and strong. 

Class averages of both str- and rls-HST showed a large reduction in the numbers of pre-powerstroke cross-bridges, Type 1, within the target zone. In iso-HST, 29% of the target zone cross-bridges were pre-powerstroke, but only 5–6% were pre-powerstroke following the length transient. The disappearance of pre-powerstroke weak-binding cross-bridges was explained by a recent kinetic model for actomyosin interactions in the muscle [98]. The number of strong-binding cross-bridges was largely unchanged, although there were fewer following the release as might have been predicted from the lower tension developed at the point of freezing. After the length transient, when 2-headed cross-bridges were observed, both heads were strongly bound; in iso-HST some 2-headed cross-bridges had a weakly bound head. The number of 2-headed, strong-binding cross-bridges increased following the stretch and decreased following the release. An increase in 2-headed strong-binding cross-bridges had previously been proposed as an explanation for changes in the X-ray diagram of the vertebrate striated muscle following a stretch [99].

Outside of the target zone, changes were less dramatic. Changes in TM bridges were small. Comparatively more TM bridges are found after a stretch, and fewer after a release. The other type of weak-binding cross-bridge, the Tn bridges, are more frequent after a release and less frequent after a stretch. Following a stretch, one strong-binding class average was found just outside of the target zone on the M-ward side, but it represented a small fraction of all strong-binding heads. Changes in TM and Tn bridges, though small, are consistent with a role in an active contraction. A stretch would be equivalent to a change toward an earlier stage of shortening, where more cross-bridges need be positioned to bind the target zone and move it M-ward thus shortening the sarcomere length. A release would be equivalent to a change toward later in the contraction where further shortening is small. If Tn bridges play a role in stretch-activation, an increase in their number would be expected toward the end of the shortening cycle. 

Changes in the lever arm axial angles of strong-binding cross-bridges were more towards rigor following a release and more towards anti-rigor following a stretch, but the differences were not large, probably reflecting the elapsed time following the length transient. Azimuthally, strong-binding cross-bridges following a length transient reflected the same highly biased lever arm distribution described above for iso-HST. 

Asynchronous flight muscles like those in *Lethocerus* are designed to oscillate rapidly so that the amount of shortening per half sarcomere is small. The muscles generally have very short I-bands consistent with a small amount of shortening. The amount of shortening varies between species but values typically range from 1–5% [100,101,102,103,104]. With a half sarcomere length of 1.3 µm in *Lethocerus*, the shortening of 3% equates to a filament sliding of 39 nm/half sarcomere, a value matching the thin filament half repeat [100]. Examined from the standpoint of a single thin filament, the shortening of 39 nm would require that a target zone be relayed between two or more successive crowns on the thick filament [97]. The relay mechanism implied by the presence of TM bridges binding M-wards of the target zone would be consistent with such a mechanism. 

Wray’s match-mismatch hypothesis [105] was offered as an explanation for the rapid, stretch-activated contractions in the *Lethocerus* flight muscle. The precise filament geometry and arrangement in the *Lethocerus* flight muscle was key to controlling the tension increase and subsequent decrease as the thick and thin filaments slide past each other. The amount of filament sliding between regions of maximal and minimal match between target zones and thick filament origins was ~19 nm, about right for 39 nm of shortening/contraction. The validity of Wray’s model has been challenged based on observations about the thick filament arrangement in the *Lethocerus* flight muscle myofibrils [106]. Oval profiles in the M-band region where myosin heads are absent suggested that thick filaments across the A-band are distributed over three azimuthal arrangements that differ by ±60º. If this propagated into the A-band, then the zones of optimal match/mismatch would be distributed over three different offsets that would result in a flat probability distribution overall. The subsequent publication of an OSR of the relaxed *Lethocerus* flight muscle [11] mentioned above, which encompassed an area of ~2.3 µm^2^, showed that the myosin crowns are aligned both axially and azimuthally over all or a large part of each half sarcomere.

### Impact of Recent Results on the Interpretation of Frozen Active Muscle Imaging

The high-resolution reconstruction from *Lethocerus* thick filaments [6] showed myosin heads arranged in an IHM oriented perpendicular to the thick filament axis rather than roughly tangential to it as found in other striated muscles (Figure 2). The blocked head was comparatively poorly ordered, but visible in the reconstruction. The helical angle of 33.98° is the largest so far observed for *Lethocerus* thick filaments. A subsequent reconstruction from filaments with poorly ordered heads showed a 0.16° reduction in the helical angle between crowns and complete disorder in the blocked-head MD, but the free head was relatively unchanged [107]. These results impact models for contracting the *Lethocerus* muscle in several ways.

1. An X-ray fiber diffraction of the relaxed *Lethocerus* flight muscle shows that tension applied sinusoidally causes changes in the helical angle between crowns as well as changes in the 14.5 nm meridional reflection. Under tension, relaxed fibers showed a 33.75° helical angle, whereas fibers under low or no tension showed a 33.90° helical angle. The changes in the 14.5 nm meridional intensity are open to interpretation, but could be signaling a change in the IHM resulting from applied tension.
2. If tension applied to the relaxed muscle can disorder the IHM, thereby producing a change in the helical angle, a conjecture at this point, then ordering the IHM in isolated filaments should increase the helical angle, which is what is observed. In other words, the rod structure, which defines the filaments helical structure, and the IHM structure appear to be coupled.
3. If only the blocked head becomes disordered during a stretch, an ironic statement given its name, the retained order in the free head pins the S2 tether to the backbone thereby restricting the “detached” blocked head to movements about the head-rod junction. Highly restricted movements of the blocked head would require a rather precisely placed target zone actin subunit to initiate force production.
4. Reformation of the interacting heads motif after a contraction must be fast enough to sequester the myosin heads from further interaction with the thin filament. The kinetics of ATP cleavage in the *Lethocerus* myosin in this context have been discussed previously [6], indicating that the ATPase speed is sufficient. However, as noted above (Figure 5), the post rigor, myosin•ATP structure of the free head might be capable of reattaching the thick filament backbone even before ATP cleavage has occurred. Free-head rebinding to the thick filament backbone would be a first order reaction and potentially faster than the ATP cleavage step that recocks the lever arm because it does not involve a covalent bond cleavage.

This idea has yet to be tested against the rich X-ray diffraction pattern of contracting *Lethocerus* flight muscle. The orientation of the IHM in the *Lethocerus* flight muscle and the filament separation means that large radial movements of blocked heads are not necessary to contact the target zone or the Tn complex [107,108]. Azimuthal movements may be more important. We note that in the work described above, azimuthal changes in the lever arm are consistently required to fit myosin head crystal structures into 3D images of actin-myosin interactions in situ. If IHMs reform after each contraction, free head rebinding to the thick filament backbone could highly restrict blocked head rebinding to the thin filament thereby contributing to shortening deactivation.

## 8. Results from Fast Frozen Vertebrate Muscle Fibers

Several differences between the vertebrate muscle and *Lethocerus* flight muscle are worth keeping in mind. The filament arrangement in the vertebrate striated muscle is much less favorable for thin section EM, particularly for viewing of longitudinal sections. The filament arrangement in flight muscle places the thin filament between two thick filaments, i.e., the 100 lattice planes, in which cross-bridges approach the thin filament from only the two neighboring thick filaments (Figure 1A). The most favorable orientation for cross-bridge viewing in the vertebrate muscle is a section cut parallel to the 110 planes of the hexagonal lattice (Figure 1B), which places two thin filaments between successive thick filaments, but the arrangement permits cross-bridges to approach and bind the thin filament from both the front and back sides of the section. 

Between relaxed and active contractions in *Lethocerus*, the thick filament axial repeat changes by only 0.05% [96] and is generally scaled to 14.5 nm without noticeable error. In the activated and rigor vertebrate muscle, the relaxed axial period of 14.3 nm increases to 14.5 nm, an increase of 1.4% [93,94]. Measurements of the rigor and contracting muscle rescaled assuming the myosin meridional has a spacing of 14.3 nm have introduced a 1.4% error. 

The F-actin period and the Tn period of the *Lethocerus* flight muscle are congruent, which results in target zone binding by myosin enhancing the inner parts of the 19.2 nm layer line [59], but in the vertebrate muscle they are not. Thus, two types of target zones could be observed. If myosin head binding were defined by actin azimuth alone, it would enhance either or both of the 36-37 nm layer line and the inner parts of its second order at 18–18.5 nm. (Changes in the TM position would affect the outer parts of the second layer line.) Enhancement of the inner parts of a 19.2 nm layer line would imply binding to target zones defined by the Tn position. 

The first application of fast freezing and freeze substitution to active the muscle utilized a rabbit psoas muscle held in a tension transducer to measure tension followed by the exchange from a rigor solution to an activating solution with ATP and calcium, which was frozen by smashing into a copper mirror cooled to liquid helium temperature [24]. Myosin head distribution in the frozen active fibers was distinctly different from rigor fibers in that heads were largely perpendicular to the fiber axis, instead of showing the characteristic arrow head appearance of rigor cross-bridges. Intensity enhancement of the 37 nm layer line corresponding to the thin filament half repeat was seen in both rigor and active fibers, but not in relaxed fibers, consistent with heavy myosin head decoration of the thin filament in the active muscle. The 14.3 nm meridional was present in active and relaxed fibers, but missing in rigor fibers indicating that the myosin heads retained their origin on the thick filament in the active muscle. An 18.5 nm layer line at low radius from the meridian was noted but not commented on. 

Hirose and Wakabayashi [85] investigated the frozen, isometrically contracting, rabbit psoas muscle utilizing similar techniques to Tsukita and Yano, but notably examining thin sections cut transverse to the filament axis. Rigor cross-bridges appeared triangular in longitudinal sections with a tilted appearance, a large contact on the thin filament and a narrow attachment to the thick filament. Few active cross-bridges had a triangular shape; most had uniform width between thick and thin filaments. Their average axial angle was close to 90°. They classified visually the different cross-bridge forms observed in transverse sections and found that rigor cross-bridges were predominately bent and active cross-bridges remarkably straight, similar to the observations described above for the *Lethocerus* flight muscle. Target zone marking was not observed in active contraction, though it was observed in their rigor images.

Hirose et al. [109] improved on this work by replacing the solution exchange with the flash photolysis of caged ATP. Notably, the fibers maintained straight Z-lines and M-lines when using cage ATP. After flash photolysis, fibers were frozen after 20, 50, 80 and 300 ms. A weak 19 nm layer line was reported in power spectra from micrographs of fibers following ATP photolysis, which might suggest the presence of target zones between successive Tn complexes marked by myosin heads. At a 20 ms time point following photolysis, some rigor cross-bridges identifiable by their 2-headed appearance are seen but none are seen after 50 ms. Note that in vertebrate thin filaments, the actin crossover spacing is 36 nm, whereas the Tn period is 38.7 nm so that, if flight muscle-like target zones were present, they would rotate +13.8° systematically every seven actin subunits along the long pitch strand of the thin filament and target zone marking would appear as an increase in intensity at 19 nm.

This work was further advanced by the application of correspondence analysis, a form of the multivariate data analysis used for the *Lethocerus* muscle, to quantitatively characterize the differences in cross-bridge shape in transverse sections [86]. By determining the direction of view of the transverse sections from serial sections, they identified three basic types of cross-bridge from sections 20 nm thick. Using the centerline connecting thick and thin filaments as the point of reference, they observed bridging density coming off the thin filament to the left or right as well as along the inter-filament axis. In rigor, most bridging density extended off the left or along the inter-filament axis, with generally a distinct bend from right to left as it approached the thick filament. Although rigor-like forms were seen at all time points after photolysis, they were lowest at 50 ms, replaced by more straightened forms that also extended off the thin filaments to the left, right or center line and with less curvature as they approached the thick filament. Although the interpretation of the results is complicated, since they are based on projections alone, one conclusion stands out clearly; active cross-bridges are different from rigor cross-bridges. 

From studies using caged ATP in a rigor solution, Lenart et al. [88] studied the response of the *Rana temporaria* (frog) sartorius muscles in a relaxing solution to release caged calcium by photolysis. The authors did not attempt to classify individual cross-bridge types, but instead examined the changes in the summed power spectrum of longitudinal sections at different time points after calcium release as well as in rigor and relaxed fibers. Many of the features known to change in X-ray fiber diffraction of the active muscle were reproduced. However, some features were apparently novel. The 36 nm layer line increases in intensity as expected, most likely due to the marking of actin subunits by myosin heads. Unexpectedly, the peak of the off-meridional intensity moved radially outward indicating that the myosin head mass was moving radially inward on the thin filament. Several observations were made about the appearance of off-meridional layer line intensity at an axial spacing of 19 nm, but the source was unknown.

A clear identification of myosin head binding to target zones in the contracting vertebrate muscle by X-ray fiber diffraction has not been shown. However, modeling studies to explain the X-ray diagram of rigor fish muscle [110] concluded that intensity increases in the 36 nm layer line could be explained by myosin head binding to target zones of 3-4 actin subunits in length, comparable to those observed for rigor *Lethocerus* flight muscle. Increases in the 36 nm layer line imply that actin azimuth is the parameter defining myosin head binding. The difference in spacing between 18.5 nm and 19.2 nm is subtile but distinguishable in an X-ray diagram from a well-ordered muscle. The target zones in *Lethocerus* shrank from 3-4 actin subunits in rigor to two subunits once nucleotide was added. A similar effect likely occurs in the vertebrate striated muscle, which would define target-zone position more precisely. 

Because the *Lethocerus* flight muscle work concentrated on 3D imaging and classification while the vertebrate muscle results generally were confined to projections and analysis of power spectra, the two sets of results are not easily compared. However, we find it intriguing that several studies observed the buildup of intensity on the inner part of the 19 nm layer line, which might indicate that the target zone marking similar to that observed in the active flight muscle and others noted azimuthal changes in the appearance of the myosin heads when compared to rigor. 

## 9. Prospects for Future Improvements

The chief limitation of the work described above on imaging actin-myosin interactions in situ is the necessity of cutting thin sections of plastic embedded tissue and improving the contrast using heavy metal stains. Ideally, the sections should be cut through the frozen-hydrated tissue and visualized without addition of heavy metals. Generally, improvements in resolution of at least a factor of 2, from 5 nm to 2.5 nm would be needed to gain any new insights into actin-myosin interactions in situ. A technique to do this, called CEMOVIS [111] has been under development for a number of years, but not yet applied to muscle tissue in any systematic way. CEMOVIS has several technical issues that remain unsolved. First, sectioning of frozen, unfixed tissue produces several artefacts, such as knife marks and crevasses [112]. These are mostly confined to one surface and are not by themselves limiting since part of the section depth appears unaffected. Second, frozen-hydrated sections suffer much more from section compression than plastic sections, which would significantly affect atomic models. Cutting single filament layers such as the myac layer is probably out of the question in the near future, but sections 100 nm thick could be cut, tilt series recorded and the tomograms “sectioned” in the computer to give single filament layer views. There is no reason in principle that the multivariate data analysis could not be used to produce class averages free of staining artefacts. In principle, CEMOVIS could be applied to smash frozen, active muscle fibers, though it may be technically challenging due to the relatively small depth of good freezing.

A more promising avenue is the application of Focused Ion Beam milling, often referred to as FIB-SEM, because the process is performed in a dual beam scanning EM [113,114,115]. In FIB-SEM, a beam of heavy ions is used to literally carve out a thin lamella in the tissue as mounted or cultured on the EM grid which can then be transferred to a TEM for subsequent tilt series data collection. The technique could be applied to a myofibril preparation spread over an EM grid. Although mechanical effects would be difficult to monitor, all of the AMPPNP induced states as well as the relaxed and rigor muscle would be accessible to this technique. Importantly, one state of active contraction, the so-called calcium poised state, in which the muscle is bathed in a MgATP solution with submaximal calcium has not been studied. Calcium poised muscle is primed to contract but requires a stretch to fully activate. Calcium poised myofibrils could yield details about how the muscle positions itself for its next contraction. Is calcium poised muscle rich in Tn bridges and weak Type I target zone attachments? There are many challenging technical issues that must be solved before active force bearing cross-bridges can be imaged in the muscle. There are still many questions remaining before a complete picture is obtained for the actively contracting muscle.

## Figures and Tables

**Table 1:** Timeline of *Lethocerus* flight muscle 3D imaging.

Year	Event	Reference
1975	3-D reconstruction from 2-D crystalline arrays	[21]
1984	3-D spatial average of *Lethocerus* rigor flight muscle from tilt series	[22]
1984	Oblique Section 3-D reconstruction of the vertebrate M-band	[23]
1985	Freeze substitution of fast frozen active vertebrate skeletal muscle	[24]
1989	Addition of thick section data to rigor tilt series 3-D reconstruction	[25]
1991	Oblique section 3-D reconstruction of rigor *Lethocerus* muscle	[26]
1993	Crystal structure of a myosin head	[27]
1994	Oblique section 3-D reconstruction of *Lethocerus* muscle in AMPPNP	[28]
1994	Automated data collection for tilt series images	[29]
1997	Dual axis tilt series tomogram of rigor *Lethocerus* flight muscle	[30]
1999	Subvolume classification of *Lethocerus* flight muscle tomograms	[31]
2001	Atomic models for rigor *Lethocerus* flight muscle cross-bridges	[32]
2004	Tomogram of frozen, freeze substituted rigor *Lethocerus* flight muscle	[33]
2010	Tomogram of frozen, freeze substituted, active *Lethocerus* flight muscle	[34]

## Abbreviations

OSR	Oblique Section Reconstruction
Tn	Troponin
TM	Tropomyosin
RLC	Regulatory Light Chain
ELC	Essential Light Chain
myac	A thin, 25 nm thick longitudinal section containing alternating myosin and actin filaments
LMM	Light meromyosin; the last ~100 nm of myosin coiled-coil rod domain
MD	Motor Domain
IHM	Interacting Heads Motif
cryoEM	Cryogenic Electron Microscopy
S1	The component of myosin containing the motor domain, light chains and lever arm
S2	Initial ~50 nm of the myosin coiled-coil rod domain
ET	Electron Tomography
AMPPNP	adenylyl-imidodiphosphate, a non-hydrolysable analog of ATP
HST	High Static Tension, i.e., an isometric contraction
